# Perceptions and viewpoints on proceedings of the Fifteenth Assembly of Heads of State and Government of the African Union Debate on Maternal, Newborn and Child Health and Development, 25–27 July 2010, Kampala, Uganda

**DOI:** 10.1186/1753-6561-5-S5-S1

**Published:** 2011-06-13

**Authors:** Luis Gomes Sambo, Joses Muthuri Kirigia, Georges Ki-Zerbo

**Affiliations:** 1 World Health Organization, Regional Office for Africa, B.P. 06, Brazzaville, Congo

## Abstract

**Background:**

Out of 358000 maternal deaths that occurred globally in 2008, 57.8% occurred in continental Africa. Africa had a maternal mortality ratio of 590 compared to 14 in developed regions, 68 in Latin America and Caribbean, and 190 in Asia. This article reflects on the discussions held during the Fifteenth Assembly of the Heads of State and Government of the African Union on the reasons why the maternal mortality ratio is so high in Africa and what can be done to reduce it.

**Methods:**

Methods employed included panel and open public discussions among the Heads of State and Government of the African Union. The article uses the WHO health systems strengthening framework, which consists of six pillars (information systems, leadership and governance, health workforce, financing, and medical products, vaccines and technologies, and health services) to describe the proceedings of the discussions.

**Discussion:**

The high maternal mortality ratios in countries were attributed to weak national health information systems; leadership and governance challenges related to poverty, health illiteracy, poor transport networks and communications infrastructure, risky cultural practices, armed conflicts and domestic violence, dearth of women empowerment; inadequate levels of skilled birth attendants; inadequate domestic and external funding; stock-outs of consumable inputs; and limited coverage of maternal and child health interventions.

In order to accelerate progress towards MDGs 4 and 5, the Heads of State and Government recommended that countries should make maternal deaths notifiable and institutionalize maternal death audits; develop, fund and implement policies and strategies geared at improving maternal, newborn and child health; accelerate inter-sectoral action to address the broad health determinants; increase the number of skilled birth attendants; fulfil commitment to allocate at least 15% of the national budget to the health sector and allocate adequate resources to prevent stock-outs of essential medicines and reproductive health commodities; leverage health promotion approaches to raise national awareness; and ensure that there is a health centre within a radius of four kilometres equipped to provide good quality integrated maternal, newborn and child health services.

**Conclusions:**

There was consensus among the discussants that there was urgent need to speed up actions for strengthening health systems to improve coverage of maternal, newborn and child health services; and to address broad determinants of women, newborn and children’s health for sustained improvements in health and other development goals.

## Background

*“When women and children die prematurely the future of a nation is doomed. And economic development is nothing if it is not people centred”*. Dr Ernest Bai Koroma, President of the Republic of Sierra Leone.

The Fifteenth Assembly of the Heads of State and Government of the African Union took place from the 25^th^ to 27^th^ of July 2010 in Kampala, Uganda. The theme of the Assembly was ‘Maternal, Infant and Child Health and Development in Africa’. His Excellency Dr Bingu Wa Mutharika, President of the Republic of Malawi and Chairman of the African Union, made the opening remarks on ‘Maternal, Infant and Child Health and Development in Africa’ [1].

He underscored the importance of the Assembly’s theme and timing as the countries were preparing for the United Nations High Level Summit to be held in September 2010 to review progress in achieving the Millennium Development Goals (MDGs) [2]. Dr Mutharika reminded the AU Assembly of its decision during the 14th Ordinary Session in January 2010 to ensure that in the next five years no child in Africa died of hunger or malnutrition or went to bed on an empty stomach. He added that without adequate food the problems associated with child birth and child survival would get worse. The attainment of MDGs 4 (reduce child mortality) and 5 (improve maternal health) is important for the socioeconomic development of Africa. He appealed to his colleagues to work towards significantly improving the health and welfare of mothers, infants and children by creating an environment for safe motherhood as the basis for future economic growth and advancement of the human race.

He informed the AU Assembly that the leaders of the Group of Eight (G8) during their meeting in Muskoka, Canada, 25–26 June 2010 adopted a declaration [3] calling upon developing governments to meet their primary responsibilities for socioeconomic development and global governance. The G8 countries had lamented over the unacceptably slow progress towards both MDGs 4 and 5, which has resulted in a large number of maternal and child deaths. They reaffirmed their support for efforts that significantly reduce the number of deaths among mothers, newborns and children aged below five years.

Dr Mutharika reported to the Assembly on the commitment made by the G8 countries at their Muskoka summit to mobilize up to USD 10 billion over the period 2010–2015 to support maternal, infant and child health. He added:

“*Let me emphasize that the responsibility for achieving this (MDGs 4 and 5) is not for the G8 or G20 countries alone. African governments must be in the forefront to attain this objective of reducing maternal, infant and child mortality. We must ask ourselves what contributions we will make towards this noble cause. Let Africa act and act now*” [1].

## Methods

The Assembly included panel and open public discussion among the Heads of State and Government of the African Union. The panellists consisted of His Excellency Yoweri Museveni, President of the Republic of Uganda; His Excellency Armando Guebuza, President of the Republic of Mozambique; Dr Thoraya Ahmed Obaid, Executive Director of the United Nations Population Fund (UNFPA); Professor Jeffrey Sachs from the Columbia Earth Institute; and Ms Yvonne Chaka Chaka, UNICEF Goodwill Ambassador. The discussions were moderated by Ms Zeinab Badawi of the British Broadcasting Corporation (BBC).

The opening speech was followed by a moderated discussion on three questions: Why is maternal mortality ratio so high? Presidents, how would you respond to the allegation by your co-panellists that in Africa “women are not a priority”? And what can be done to reduce maternal mortality?

In this paper we have followed the WHO health systems strengthening framework to describe the proceedings of the discussions. The World Health Report 2000 defines “a health system to include all the activities whose primary purpose is to promote, restore or maintain health” [4:p.5]. In the context of the current theme of the African Union Assembly, health systems have four specific goals: improving maternal, newborn and child health; responding to women’s and children’s non-medical expectations; providing women and children with social and financial risk protection against the costs of ill-health; and making efficient use of available resources [5].

Those goals are realized by national health systems through performance of their key functions of stewardship (oversight), creating resources (investment and training), delivering services (provision), and financing (revenue collection and pooling and purchasing of health commodities) [4]. Out of these four functions, WHO [5:p.3] proposes six building blocks of a health system:

• ***Information systems:*** Production, analysis, dissemination and use of reliable and timely information on health determinants, health systems and health status of people [5];

• ***Leadership and governance:*** Ensuring existence of an effective policy framework covering national health policy and national health sector strategic planning; coalition building, including coordination of partnerships and intersectoral action for health; appropriate regulations; and accountability [5].

• ***Health workforce:*** Availability of sufficient numbers and mix of competent, responsive, productive and fairly distributed staff engaged in actions whose primary intent is to protect and improve health [5];

• ***Financing:*** Adequate funding for health in ways that ensure women and children can access needed services and are protected from financial catastrophe that could result from using the services [5];

• ***Medical products*, *vaccines and technologies:*** Access and rational use of essential medicines, vaccines and other health technologies of assured quality, safety, efficacy and cost-effectiveness [5]; and

• ***Health services:*** Delivery of effective, safe, quality personal and non-personal preventive, curative, rehabilitative and health-promotion interventions for women, newborns and children who need them [5].

## Discussion

**Moderator:** Why is maternal mortality rate so high?

The panellists regretted that too many women’s lives were being lost in the process of “giving life” despite the availability of knowledge on the requisite effective interventions such as family planning; presence of skilled birth attendants during pregnancy, childbirth and the postnatal period; and health promotion interventions to enable individuals, families and communities to improve their awareness, knowledge and behaviours towards maternal and newborn health. The panellists attributed the high maternal mortality ratios to various factors related to weak health systems:

• **Information systems:** National information systems are too weak to accurately generate and analyse information on health status and determinants of health of women, newborns and children. An important component of information systems is the dissemination of information in formats that are adapted to local contexts.

• **Leadership and governance:** Poverty and health illiteracy contribute to households delaying the decision to take pregnant women to health facilities for delivery, and poor transport networks and communications infrastructure account for the delay to get to a health facility for delivery. Risky cultural practices such as female genital mutilation, use of herbs to induce labour, refusal of caesarean section (when medical conditions make it necessary) to prove womanhood, and certain cultural beliefs hinder pregnant women from seeking antenatal and postnatal care. Moreover, armed conflicts and domestic violence add to the vulnerability of women. Issues of women empowerment and priority setting by governments also impact women’s welfare. Within the context of leadership the State must be seen to formulate evidence informed policies for the reduction of maternal deaths. The State would also organize and regulate services. Concerning governance the participation of the users of services in the running of these services is important. The health provision services should be seen to serve populations, not themselves. There has to be a transparent management of the health systems with governments held accountable for the achievements.

• **Health workforce:** There are inadequate levels of skilled birth attendants, poor distribution of health resources with a skew towards urban vis-à-vis rural areas, and delays in receiving appropriate care at health facilities often owing to a dearth of skilled birth attendants. An important concept in the workforce is their distribution in terms of numbers and skills. One may also pay attention to the referral system- based on the skills available at the different levels of the pyramid.

• **Financing:** The panellists noted that there was inadequate domestic and external funding targeted at maternal, newborn and child health problems. Also, earmarking and donor priorities were an impediment to spending on national priorities such as maternal and child health. These constituted serious challenges for the health delivery system. In addition to these, most of the funding pledged at various G8 meetings is yet to be received by the African countries.

• **Medical products, vaccines and technologies:** Stock-outs of consumable inputs such as medicines, gloves and caesarean section kits and inadequate coverage of health infrastructure are widespread on the continent.

• **Service delivery:** Coverage of maternal and child health interventions is constrained by limited access to health facilities in some geographical regions and poor referral systems. Maternal and child health is quite often associated with specific diseases such as HIV/AIDS, malaria, tuberculosis and neglected tropical diseases, yet funding is earmarked in specific vertical programmes, which may compromise the delivery of comprehensive care at the local level.

**Moderator:** Presidents, how would you respond to the allegation by your co-panellists that in Africa “women are not a priority”?

Two of the panellists attributed the high maternal mortality ratios to the lack of prioritization of women’s health in resource allocation. Two of the presidential panellists argued that it was wrong to view investments in women’s health only through the lens of health sector spending. Instead, improvements in women’s health require a multisectoral approach. This calls for investments in education, food, water and sanitation, roads, shelter, and electricity, among others.

The panellists attributed the low investments in women’s health to the low gross domestic product in many African countries amidst other competing priorities. One of the presidents stated that “we need to start with production and see how to distribute. We can’t talk of social expenditures without production.” A panellist, representing a nongovernmental institution, responded that “If women and children are dying prematurely, who will produce? Investment in women’s health is about enhancing their already great economic contributions – since they have been the backbone of African economies.”

The Heads of State and Government vehemently rejected the notion that there was lack of political will to address the high maternal and child mortality levels.

**Moderator:** What can be done to reduce it?

After the panel discussion, the moderator asked all the Heads of State and Government present what they thought could be done to accelerate progress towards MDGs 4 and 5. The highlights of their discussion are presented below.

• **Information:** There was a recommendation to make maternal death notifiable and to institutionalize maternal death audits to ensure that causes were identified and addressed. This is already in operation in some countries such as South Africa.

• **Leadership and governance:** A number of recommendations were made for improving leadership and governance of health systems related to maternal, newborn and child health:

– In order to accelerate the implementation of the Child Survival Strategy [7], countries should develop, fund and implement evidence-based policies and strategies geared at improving newborn and child health.

– In line with the Road Map for accelerating the attainment of the MDGs relating to maternal and newborn health in Africa [8] and the Maputo Plan of Action on Sexual and Reproductive Health and Rights [9], countries should develop, fund and implement policies and strategies geared at improving maternal and newborn health.

– Countries should launch and sustain the Campaign on Accelerated Reduction of Maternal Mortality in Africa (CARMA) [10].

– The African Union Commission should ensure that annual reporting on maternal, infant and child health is a standing agenda in all future African Union meetings.

– Countries should accord maternal, infant and child health the same status as HIV/AIDS, tuberculosis and malaria in resource allocation and better ensure integration of efforts to reach MDGs 4, 5 and 6.

– In order to reduce unsafe abortions, countries could adopt laws that allow qualified health personnel to terminate unwanted or at-risk pregnancies.

– Countries should accelerate intersectoral action among health, nutrition, food security, water, sanitation, shelter, transport, and communication infrastructure geared at addressing the broad determinants of women, newborn and child health [11-13].

• **Health workforce:** Two suggestions were made on ways to ameliorate health workforce shortages. Firstly, increasing the number of skilled birth attendants through training of new midwives and on-job training of existing health workforce will improve skills to perform deliveries. Secondly, community health workers should be equipped with appropriate skills to conduct normal deliveries, as in Ethiopia.

• **Financing:**

– African leaders must take the lead in this by fulfilling their funding commitments, including that to allocate at least 15% of the national budget to the health sector [6].

– In order to improve financial access, countries should consider making health services free for pregnant women, lactating mothers and children under the age of 5 years.

– Countries should devise creative ways of raising the necessary resources to improve maternal and child health such as social health insurance and community health insurance.

– Approaches such as performance-based financing could be used to increase efficiency of resources available for addressing maternal and child health.

• **Medical products, vaccines and technologies:** Adequate resources need to be mobilized and allocated to ensure that there are no stock-outs of essential medicines and reproductive health commodities.

• **Service delivery:** A number of suggestions relating to health service delivery were made for consideration by countries:

– There is need to leverage health promotion approaches and methods to raise national awareness on the high maternal mortality problem and courses of action that need to be urgently implemented to curb it.

– Health promotion approaches should be used to increase awareness among women that it is their human right to access health services and to enjoy a good health status.

– A woman with leadership capacity should be designated in each local area to follow up pregnant women to ensure that they seek antenatal care, deliver at a health facility and receive postnatal care. Such a person could be supplied a cellular phone to call for assistance from health facilities. This kind of initiative is being implemented in Senegal.

– Countries should ensure that there is a health centre within a radius of four kilometres of all their citizens to improve access to health services.

– The primary health care approach should be leveraged to reinforce capacities of health systems to provide good quality integrated maternal, newborn and child health services.

## Conclusion

In his closing speech, President Mutharika echoed his previous appeal to African leaders to take urgent action on health of their citizens [14]:

“*You will recall that in my acceptance speech early this year in Addis Ababa I lamented the fact that our organization is very good at passing decisions and issuing declarations but has very little to show on the implementation side. I wish to repeat what I said, that the time for passing decisions and declarations is long gone and its now time for action and more action so that our people are able to see tangible benefits of having their countries as Member States of the African Union.*”

Other Heads of State and Government echoed President Mutharika’s view that since the causes and solutions [14] of the relatively high maternal, newborn and child morbidity and mortality were known, it was time for countries and the African Union to go behold passing decisions and declarations to taking bold steps to strengthen health systems and accelerate intersectoral action to address the broader determinants of women, newborn and child health. The African Heads of State and Government discussed and deliberated on maternal, newborn and child health in the continent, exercised their leadership and provided political guidance to governments, international partners and African communities on how to accelerate Africa’s progress towards MDGs 4, 5 and 6 [15]. There was consensus that it is time to speed up actions for sustained improvements in health and other development goals.

## List of abbreviations used

BBC: British Broadcasting Corporation; CARMA: Campaign on Accelerated Reduction of Maternal Mortality in Africa; MDG: Millennium Development Goal; UNICEF: United Nations Children’s Fund; UNFPA: United Nations Population Fund; WHO: World Health Organization.

## Competing interests

The authors declare that they have no competing interests.

## Authors’ contributions

LGS was a panellist in both the financing and malaria sessions. LGS, JMK and GK worked together to write the manuscript. All authors read and approved the final manuscript.
